# Parallel Selection in Domesticated Atlantic Salmon from Divergent Founders Including on Whole-Genome Duplication-derived Homeologous Regions

**DOI:** 10.1093/gbe/evaf063

**Published:** 2025-04-18

**Authors:** Pauline Buso, Célian Diblasi, Domniki Manousi, Jun Soung Kwak, Arturo Vera-Ponce de Leon, Kristina Stenløkk, Nicola Barson, Marie Saitou

**Affiliations:** IHPE, Université de Montpellier, CNRS, Ifremer, Université de Perpignan Via Domitia, Montpellier, France; Centre for Integrative Genetics (CIGENE), Department of Animal and Aquacultural Sciences, Faculty of Biosciences, Norwegian University of Life Sciences, Ås, Norway; Centre for Integrative Genetics (CIGENE), Department of Animal and Aquacultural Sciences, Faculty of Biosciences, Norwegian University of Life Sciences, Ås, Norway; Centre for Integrative Genetics (CIGENE), Department of Animal and Aquacultural Sciences, Faculty of Biosciences, Norwegian University of Life Sciences, Ås, Norway; Centre for Integrative Genetics (CIGENE), Department of Animal and Aquacultural Sciences, Faculty of Biosciences, Norwegian University of Life Sciences, Ås, Norway; Centre for Integrative Genetics (CIGENE), Department of Animal and Aquacultural Sciences, Faculty of Biosciences, Norwegian University of Life Sciences, Ås, Norway; Centre for Integrative Genetics (CIGENE), Department of Animal and Aquacultural Sciences, Faculty of Biosciences, Norwegian University of Life Sciences, Ås, Norway; Centre for Integrative Genetics (CIGENE), Department of Animal and Aquacultural Sciences, Faculty of Biosciences, Norwegian University of Life Sciences, Ås, Norway

**Keywords:** domestication, MHC, artificial selection, aquaculture, ohnolog

## Abstract

Domestication and artificial selection for desirable traits have driven significant phenotypic changes and left detectable genomic footprints in farmed animals. Since the 1960s, intensive breeding has led to the rapid domestication of Atlantic salmon (*Salmo salar*), with multiple independent events that make it a valuable model for studying early domestication stages and the parallel evolution of populations of different origins subjected to similar selection pressures. Some aquatic species, including Atlantic salmon, have undergone whole-genome duplication (WGD), raising the possibility that genetic redundancy resulting from WGD has contributed to adaptation in captive environments, as seen in plants. Here, we examined the genomic responses to domestication in Atlantic salmon, focusing on potential signatures of parallel selection, including those associated with WGD. Candidate genomic regions under selection were identified by comparing whole-genome sequences from aquaculture and wild populations across 2 independently domesticated lineages (Western Norway and North America) using a genome-wide scan that combined 3 statistical methods: allele frequencies (F_ST_), site frequency (Tajima's D), and haplotype differentiation (XP-EHH). These analyses revealed shared selective sweeps on identical SNPs in major histocompatibility complex (MHC) genes across aquaculture populations. This suggests that a combination of long-term balancing selection and recent human-induced selection has shaped MHC gene evolution in domesticated salmon. Additionally, we observed selective sweeps on a small number of gene pairs in homeologous regions originating from WGD, offering insights into how historical genome duplication events may intersect with recent selection pressures in aquaculture species.

**Significance**

Domestication rapidly alters the genomic architecture of farmed animals to enhance favorable traits, yet the early genetic responses to domestication are incompletely understood in many systems. By analyzing 2 independently domesticated Atlantic salmon lineages, this study identified shared genomic regions under selection across continents, particularly in immune-related genes. This suggests that independent domestication events can lead to similar genetic changes under artificial selection, highlighting the role of immune-related genes in Atlantic salmon evolution.

## Introduction

Domestication marks one of the key shifts in human history, tracing back to the enduring bond between early hunter-gatherers and wolves over 15,000 year ago (Vigne 2011). Domesticated plants and animals became essential to human societies for social, nutritional, symbolic, or economic purposes, reflecting the development of multicultural societies that emerged at the end of the last ice age (Larson et al. 2014). Domestication refers to a small, selected fraction from a wild population adapting to a human-controlled environment. This process can change the genetic composition and phenotypes of farmed individuals in response to both intentional and unintentional selection (Wang et al. 2014). Small initial founder populations result in a strong bottleneck and limited genetic diversity. Consequently, the genomic evolution of domesticated species is influenced by strong genetic drift, as well as intentional artificial selection for favorable production traits (Innan and Kim 2004), and unintentional domestication selection resulting from adaptation to the captive environment and human contact (Mignon-Grasteau et al. 2005; Frantz et al. 2020; Huang et al. 2022). Thus, domesticated species carry signatures of these processes in their genomes, providing valuable insights into their domestication history and make them good models for genetic studies of evolution (Wiener and Wilkinson 2011).

Conventional investigations into the evolutionary changes that occurred after domestication began with archaeological records (Diamond 2002). Recent advances using Omics approaches have enabled researchers to trace the geographical and temporal origins of domestication and its early stages. These approaches have revealed the genomic basis of favored traits and selected loci in various species (Frantz et al. 2020), while also shedding light on the unintended consequences of these processes (Glover et al. 2017; Fernandez et al. 2021; Izawa 2022). Domestication processes are often associated with selection pressures on key production traits such as growth, immunity, and age of sexual maturity (Gjederm 1979; Milla et al. 2021; Podgorniak et al. 2022). Artificial selection for such traits and domestication selection may lead to parallel evolution between independent populations exposed to similar selection pressures. This refers to the independent development of genetic adaptations within different populations that affect the organism's phenotype in the same way (Wood et al. 2005; Bailey et al. 2017). For instance, high-altitude adaptation has been observed within independent human populations and their domesticated animals such as dogs, cows, horses, sheep, pigs, and chickens, highlighting a shared genetic response to environmental pressures (Witt and Huerta-Sánchez 2019). Similarly, mammals that have adapted to high-starch diets, including various domesticated animals, have an increased copy number of the amylase gene (*amy*) (Pajic et al. 2019).

Compared to the long history of terrestrial livestock domestication, the domestication of most aquaculture species is relatively recent. Finfish aquaculture expanded rapidly in the early 1980s, while domestication timelines vary across aquaculture species (Houston et al. 2020; Teletchea 2021). Unlike established terrestrial livestock species, aquaculture species present a varied landscape of domestication success, with some species achieving breakthroughs in captive breeding while others continue to rely on wild stock imports. Successful domestications in aquaculture systems are often observed in certain fish families, such as sturgeons, cyprinids (including carp), and salmonids. Notably, some of these families have undergone whole-genome duplication (WGD), which has been linked to increased genomic redundancy, allelic diversity, heterozygosity, and meiotic recombination (Lien et al. 2016; Gundappa et al. 2022). These genomic features may be one factor to enhance tolerance to new mutations and facilitate adaptation to human-mediated environments, while WGD is not essential for successful domestication (Salman-Minkov et al 2016; Baduel et al. 2019; Mihajlovic et al. 2025; Wang et al. 2024). Many successful aquaculture species, including catfish, sea bass, and flatfishes, among others, have achieved domestication without lineage-specific WGD events. Beyond fish, domesticated aquaculture species also include mollusks (e.g. oysters and mussels) and crustaceans (e.g. shrimp and crabs), which do not have known histories of lineage-specific WGD (Houston et al. 2020; Naylor et al. 2021). While WGD-derived genetic redundancy may still contribute to adaptation in some lineages, multiple genomic pathways facilitate domestication across diverse taxa.

The Atlantic salmon (*Salmo salar*) genome underwent a WGD event (Ss4R) ∼100 million years ago, leading to the retention of a substantial fraction of duplicated genes (Macqueen and Johnston 2014; Gundappa et al. 2022). Unlike a random loss of duplicate genes, rediploidization followed a structured trajectory, resulting in a nonrandom spectrum of ohnolog divergence levels. These ohnologs are organized into collinear blocks across the genome, reflecting the evolutionary history of this process (Lien et al. 2016; Robertson et al. 2017). In this study, we use “homeolog” to refer to corresponding genomic regions and “ohnolog” to refer to duplicated genes arising from WGD events. While many duplicated genes were subsequently lost, a considerable proportion has been maintained as ohnolog pairs, contributing to functional diversification. Some ohnologs retained their ancestral function, while others underwent subfunctionalization or neofunctionalization, leading to functional differentiation and specialization. In addition to structural retention, the expression of Ss4R-derived ohnologs has evolved under selective pressures, leading to differential expression patterns. Some ohnolog pairs exhibit complementary expression across tissues, while others have diverged functionally (Lien et al. 2016). Additionally, understanding these expression dynamics is crucial for interpreting how Ss4R-derived variation contributes to domestication and aquaculture adaptation, particularly in traits related to metabolism, immune function, and stress response.

Among the aquaculture species with duplicated genomes, Atlantic salmon, has been subject to strong artificial selection and has seen growing economic importance since the 1960s (Cross and King 1983; Ford and Myers 2008; Glover et al. 2017). Atlantic salmon is a widely distributed anadromous species characterized by a strong tendency to reproduce in the rivers of their birth, which encourages genetic divergence among populations (Klemetsen et al. 2003; Lehnert et al. 2019). Wild populations of Atlantic salmon have been split into 4 main phylogeographical lineages, 3 in Europe (East Atlantic, Barents/White Sea, and Baltic) and 1 in North America (West Atlantic) (Bourret et al. 2013; Rougemont and Bernatchez 2018). These lineages exhibit karyotypic variation, with the North American populations displaying a polymorphism in chromosome numbers due to fusions (Brenna-Hansen et al. 2012). Atlantic salmon in Europe and North America have been independently domesticated multiple times from different source populations, with repeated artificial selection directed at similar traits under human-induced environments. This suggests that parallel evolution has likely occurred in domesticated Atlantic salmon populations across both continents, driven by comparable selective pressures.

Despite its recent domestication, studies of Atlantic salmon have revealed rapid genetic changes in response to artificial selection targeting metabolism, disease resistance, and faster growth (Debes et al. 2012; Bicskei et al. 2014; Harvey et al. 2018; Houston and Macqueen 2019; Jin et al. 2020; Bull et al. 2022). Moreover, signatures of domestication selection have also been observed in neurological genes (Bertolotti et al. 2020), causing behavioral alterations, such as increased sensitivity to predation and increased tolerance to stress compared with the wild (Solberg et al. 2020). Especially, the probability of parallel evolution is dependent on the richness of genetic variation, standing genetic variation when this occurs over short time periods (Wood et al. 2005; Bailey et al. 2017; Ebadi et al. 2023). Several studies have investigated parallel selection in Atlantic salmon across different geographic lineages. López et al. (2019) observed parallel evolution at 4 genes, e.g ubiquitin-conjugating enzyme E2F putative (*ube2f*), collagen alpha-1XIII chain (*coda1*), autism susceptibility 2 protein-like (*auts2-like*), and transient receptor potential cation channel subfamily M member 3-like (*trpm3-like*) between Scottish and North American populations (i.e. Canada). However, Naval-Sanchez et al. (2020) did not observe any overlap among selected loci between populations of European and North American origin, highlighting the need for further research to clarify the role of parallel selection in Atlantic salmon domestication.

Here, we investigated the genomic responses of independent Atlantic salmon lineages to recent domestication and artificial selection. By examining genomic divergence between wild and aquaculture populations in Western Norway and North America, we aimed to: (i) identify loci and pathways involved in the early stages of domestication, (ii) explore parallel evolution across lineages, and (iii) assess the impact of the salmonid-specific WGD on domestication responses. Using whole-genome sequencing and multiple selections scan methods (F_ST_, Tajima's D, and XP-EHH), we identified loci under selection, including shared loci between the 2 lineages, with a few examples of parallel selection on ohnolog pairs (i.e. genes retained in duplicate from the salmonid-specific WGD).

## Results

### Population Genetic Structure

**Fig. 1.**
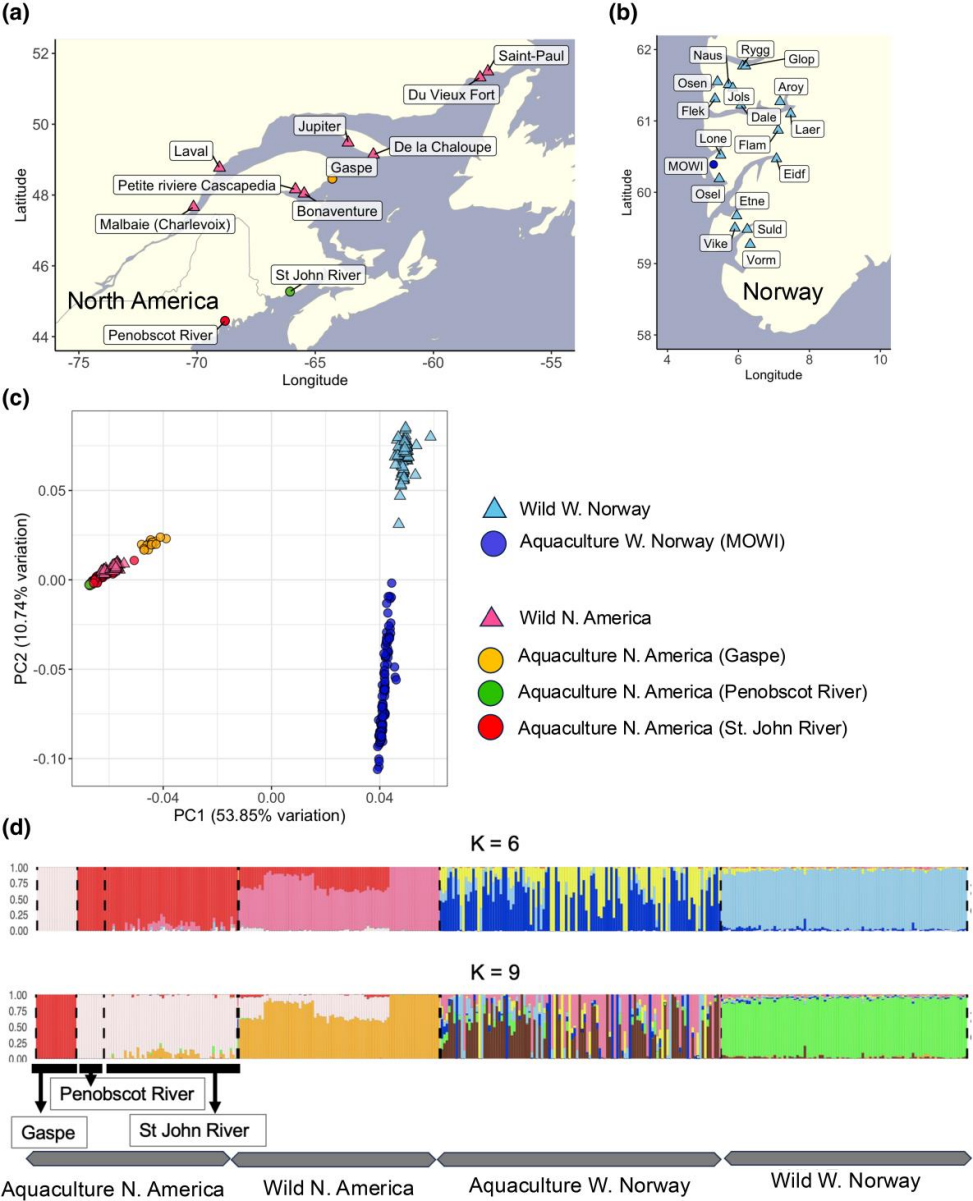
Sample distribution and genetic structure of Atlantic salmon lines from western Norway and North America. a) Geographic distribution of Atlantic salmon population in North America. Aquaculture samples were used from Gaspe (Gaspe, *n* = 16), St John River (*n* = 53), and Penobscot River (*n* = 11). Wild samples came from 8 rivers: Malbaie Charlevoix (*n* = 10), Bonaventure (*n* = 10), Petite riviere Cascapedia (*n* = 10), Laval (*n* = 10), De la Chaloupe (*n* = 10), Jupiter (*n* = 10), Du Vieux Fort (*n* = 10), and Saint-Paul (*n* = 10). b) Geographic distribution of Atlantic salmon population from Western Norway. Aquaculture samples come from MOWI Norwegian breeding line (*n* = 112). Wild counterparts were collected among 17 rivers: Vorma (Vorm, *n* = 2), Suldalslågen (Suld, *n* = 10), Vikedalselva i Vindafjord (Vike, *n* = 10), Etneelva (Etne, *n* = 2), Eidfjordvassdraget (Eidf, *n* = 2), Oselva i Os (Osel, *n* = 2), Loneelva i Osterøy (Lone, *n* = 2), Flåmselva (Flam, *n* = 10), Lærdalselva (Laer, *n* = 2), Årøy (Aroy, *n* = 10), Daleelva (Dale, *n* = 2), Flekkeelva (Flek, *n* = 2), Nausta (Naus, *n* = 10), Jølstra (Jols, *n* = 10), Oselvvassdraget (Osen, *n* = 10), Ryggelva (Rygg, *n* = 10), and Gloppenelva (Glop, *n* = 2). c) Scatterplot of PCA. PC1 and PC2 were calculated for all Atlantic salmon samples. d) Population genetic structure analysis. All samples were clustered into 6 and 9 genetic components (*K* = 6 and *K* = 9) according to the lowest cross-validation error.

We first examined the genetic structure of the sequenced populations. We compared the genetic patterns of aquaculture samples from MOWI (Norwegian) with their wild counterparts from 17 rivers in West Norway, the region from where this domesticated lineage originates. Similarly, we analyzed aquaculture samples from 3 independently domesticated North American lineages and wild samples from 8 rivers in the Gulf of St. Lawrence for comparison (Fig. 1a and b). Principal component analysis (PCA) (Fig. 1c) based on 148,433 SNPs derived from whole-genome sequencing showed 4 distinct clusters: (i) Norwegian aquaculture, (ii) wild Norwegian, (iii) North American aquaculture from the St John and Penobscot rivers and wild North American, and (iv) North American aquaculture from Gaspe. The first 2 principal components (PCs) jointly accounted for 64.59% of the total variance, with PC1 (53.85% of variance) separating the East Atlantic (North American) and West Atlantic (West Norwegian) lineages, and PC2 (10.74%) distinguishing between wild and aquaculture populations in Norway, while we did not observe a clear separation between wild and aquaculture populations in North America (supplementary fig. S2, Supplementary Material online). In agreement with the PCA, ADMIXTURE analysis (Alexander et al. 2009; Alexander and Lange 2011) also highlighted a clear separation between the 2 geographical regions. At *K* = 6, where the cross-validation error plateaued (supplementary fig. S3, Supplementary Material online), each phylogeographical lineage showed distinct ancestry, with varying extents of shared ancestry among wild and aquaculture individuals from the same lineage (Fig. 1d). Despite recent domestication from wild populations (Gjedrem et al. 1991; Glover et al. 2009; Bradbury et al. 2022), the Norwegian aquaculture population demonstrated higher genetic heterogeneity than its wild counterpart, possibly reflecting the admixed origin from multiple wild populations. Conversely, North American aquaculture and wild populations were genetically more similar, except for the Gaspe individuals, which were largely distinct, with limited representation in wild populations. This reflects more recent coancestry among North American individuals resulting from the later development of aquaculture in Eastern North America. We also observed a small amount of admixture from the wild East Atlantic group in both wild and aquaculture North American populations, reflecting known past hybridization events that have occurred both through natural secondary contact and as a result of escaped aquaculture individuals with admixed European and North American ancestry (Bradbury et al. 2022).

We delineated our study populations into 4 main groups for the following analysis: West Norwegian Aquaculture Group, Wild West Norwegian Group, North American Aquaculture Group, and Wild North American Group. This grouping was based on the tight PCA clustering of all North American populations and the smaller variance among wild Norwegian populations on PC2 than within the MOWI aquaculture strain. The groups, therefore, reflect both the continent-scale geographical origin, Western Norway versus North America, and the selective environment, those that are wild versus those that have undergone domestication.

### Identification of Putatively Selected Regions, Genes, and SNPs

**Fig. 2.**
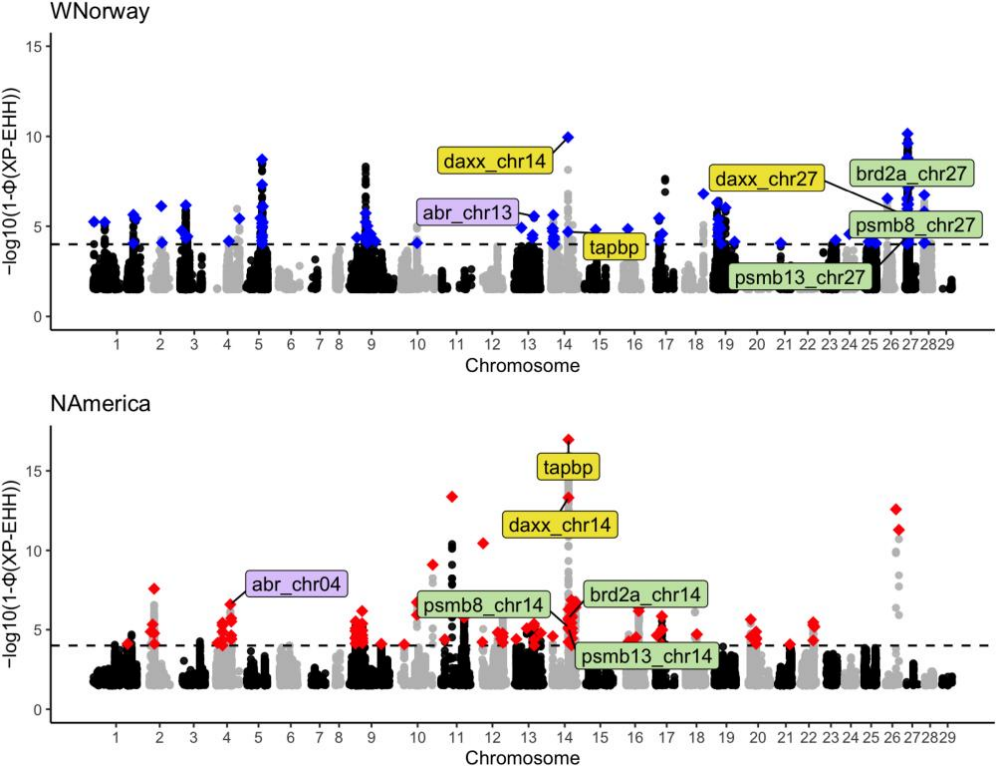
Cross-population test on extended haplotype homozygosity (XP-EHH). Each dot is SNPs, while the *x* axis displays the position across the genome. The *y* axis represents the −log10(*P*-value the XP-EHH (cross population extended haplotype homozygosity) values. Diamonds indicate SNPs with significantly high XP-EHH values that overlap genes in aquaculture populations from Western Norway and North America, respectively. We adopted −log10(*P*-value) = 4 as the cutoff (dashed line) for significant XP-EHH values (Materials and Methods). Gray and black dots are other tested SNPs. Only SNPs with a -log10(*P*-value) >1 were plotted to ensure the computational efficiency. Labeled genes are genes with exact same SNPs under parallel selection (yellow) or selection on the homeologous regions, with matching colors in both populations showing the WGD-derived gene pairs. The suffix denotes the chromosomal location of the copy. The entire SNP list with significant XP-EHH is available in supplementary table S1, Supplementary Material online.

The genome scans to identify potential genomic regions under artificial selection in domestic strains were carried out on 1,139,312 SNPs for Tajima's D and F_ST_ and 1,053,324 SNPs for XP-EHH, depending on the filtering requirements (see Methods). Tajima's D revealed 1,053 windows in the Norwegian aquaculture population and 1,032 windows in the North American aquaculture populations that are putatively under selection. Regarding F_ST_, the analysis identified 3,177 windows in the West Norwegian pair and 3,088 in the North American pair as potentially under divergent selection. Finally, XP-EHH identified 1914 SNPs in Western Norway and 1982 in North America that are under putatively recent directional selection (Fig. 2).

We then asked if there were any regions or SNPs suggested as under selection by multiple methods. For the pairs in Western Norway and North America, we identified 8 and 2 windows, respectively, where regions were suggested to be non-neutral by both the F_ST_ and Tajima's D metrics. Similarly, there are 104 and 339 shared SNPs suggested to be non-neutral by both F_ST_ and XP-EHH in Western Norway and North America, respectively. Meanwhile, no SNPs overlapped between Tajima's D results and XP-EHH results on either continent. The limited overlap among loci detected as outliers between different methods could be because each method is sensitive to detecting selection over different timescales (Oleksyk et al. 2010). Lack of overlapped loci among methods has been reported previously for domesticated Atlantic salmon (López et al. 2019).

To assess the statistical significance of overlapping genomic sweep regions identified by multiple metrics, we performed random size-matched window sampling simulations (*n* = 1000). For Tajima's D and F_ST_ comparisons, the observed overlap fell below the bottom 1% of the simulated distribution. In contrast, for XP-EHH and F_ST_, the observed overlap exceeded the maximum value in the simulations (supplementary fig. S4, Supplementary Material online). These findings suggest that Tajima's D and F_ST_ capture selection signatures at different evolutionary timescales, leading to lower-than expected overlap, whereas XP-EHH and F_ST_ tend to highlight regions where selective sweeps and resulting population differentiation cooccur.

To infer which functional groups of genes have been subjected to selection, we identified genes that overlap with windows or SNPs suggested to be non-neutral by multiple methods, using the genes from the Ensembl v111 annotation of the Atlantic salmon reference genome. For these genes, we conducted Gene Ontology (GO) enrichment analysis (Table 1). The “Focal adhesion (false discovery rate, FDR = 0.015)” category has been detected in Western Norway from the intersect F_ST_/Tajima's D. No GO categories were detected in other groups of genes detected by multiple methods. Therefore, we expanded our investigation to include results from XP-EHH alone, which can detect higher resolution and more recent directional selection. For the genes that contain SNPs identified by XP-EHH, the categories “semaphorin receptor binding (FDR = 0.01)” and “organelle organization (FDR = 0.013)” were enriched in Western Norway, while the category “intracellular protein-containing complex (FDR = 0.0077)” was enriched in North America. A complete list of genes overlapping with XP-EHH outliers is available in Supplementary material (supplementary table S3, Supplementary Material online).

**Table 1:** GO enrichment results on genes under putative selection

FDR	nGenes	Fold enrichment	Pathways	Population	Tests
0.015	3	13.8	Focal adhesion	W. Norway	Intersect F_ST_/Tajima D
0.01	4	27.8	Semaphorin receptor binding	W. Norway	XPEHH
0.013	14	3.6	Organelle organization	W. Norway	XPEHH
0.0077	6	11.2	Intracellular protein-containing complex	N. America	XPEHH

For genes that overlap with windows or SNPs suggested to be non-neutral by multiple methods, or XPEHH alone, we conducted GO enrichment analysis using ShinyGo v0.77 (Ge, Jung and Yao 2020). Categories are shown when the enrichment FDR was < 0.05, Fold Enrichment > 2, and the GO category contained ≥ 3 our input genes (nGenes). FDR is adjusted from the hypergeometric test. Fold Enrichment is defined as the percentage of genes in the input list belonging to a pathway, divided by the corresponding percentage in the background genes (Ge, Jung and Yao 2020).

### Parallel Selective Sweeps on the Shared SNPs in the MHC Locus in 2 Atlantic Salmon Lineages

**Fig. 3.**
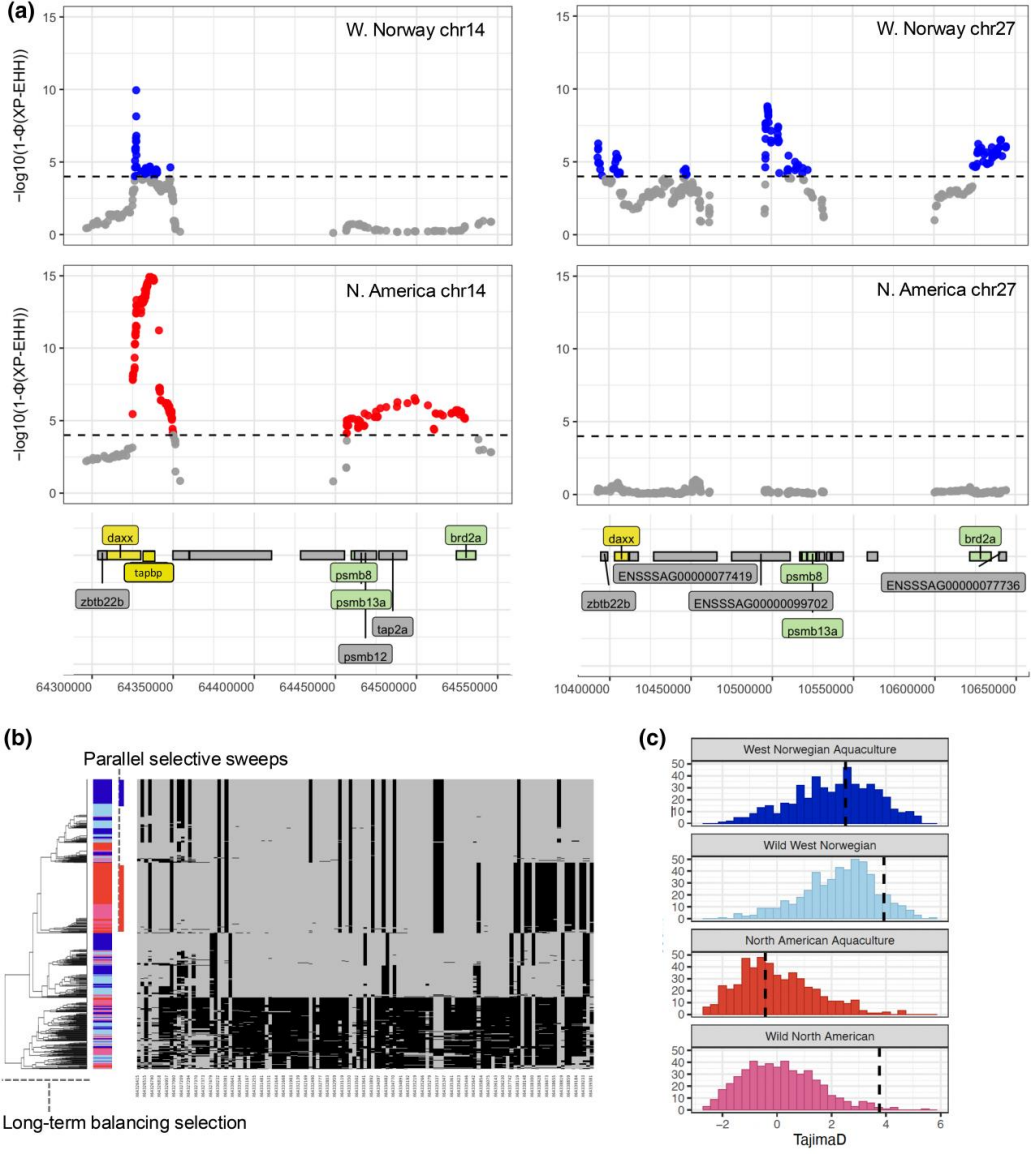
The MHC region under parallel selective sweeps in both continents. a) Zoomed-in view of XP-EHH results on the MHC loci on chromosomes 14 and 27, exhibiting parallel selective sweeps. The *x* axis shows the genomic position, and the *y* axis represents the −log10(*P*-value) of the XP-EHH (cross population extended haplotype homozygosity) values. Candidate SNPs with significantly high XP-EHH values (above the threshold −log10(*P*) = 4, indicated by the dashed line) are shown, with matching SNPs across populations indicating parallel selection signals. Genes under parallel selection or in homeologous regions with shared selective signals are highlighted accordingly, and genes with signals specific to one population are also labeled. b) Haplotype pattern in the MHC region (126 SNPs from chr14: 64326415-64339381), with each column representing a SNP and each row a phased haplotype from the four population groups. The group is labeled accordingly. c) Distribution of Tajima's *D* values for each population, shown as histograms. The vertical dashed line indicates the observed Tajima's *D* value at the MHC locus. Neutral expectations are derived from 500 randomly sampled size-matched regions per population.

We assessed parallel genomic patterns by comparing candidate positively selected XP-EHH SNPs of aquaculture groups across continents, as this method was likely to be most specific to recent divergent selection. We found 82 SNPs with extended haplotypes in both aquaculture lineages (Fig. 3a). Of these, 58 SNPs fell within the *tapbp* gene (ENSSSAG00000040723, 14:64331687-64338464) and the rest, 24 SNPs fell within the adjacent *daxx_chr14* gene (ENSSSAG00000040701, 14:64304883-64330023) on chromosome 14. Both genes are located into the major histocompatibility complex (MHC) class IA region. Notably, 5 of the shared SNPs are missense SNPs. To check if this result reflected genotyping errors owing to the high diversity and duplicated nature of the MHC region, we manually inspected the SNPs using the BAM files in IGV (Robinson et al. 2011). We assessed that these SNPs are likely real (supplementary fig. S6, Supplementary Material online) and are reliably genotyped.

Variation in the MHC region is known to be maintained by long-term balancing selection as observed as trans-species polymorphisms (Tsukamoto et al. 2012; Teixeira et al. 2015). Given that variation in the MHC region has been maintained since before the divergence of the lineages, we would expect high Tajima's D values in this region. To investigate it, we conducted haplotype analysis and calculated Tajima's D on this MHC region on chromosome 14 and random, size-matching 500 control regions in the 4 groups. Haplotype clustering using the 125 MHC SNPs on chromosome 14 (chr14:64326415-64339381) suggested that there are 2 independent selective sweeps in the West Norwegian aquaculture group and North American Aquaculture group and that high diversity in the haplotypes has been maintained in Atlantic salmon since before the lineage divergence between the lineages of North America and Europe (Fig. 3b). In the wild North American group, the Tajima's D value for this region was at the 1st percentile compared to the control regions, while the Tajima's D value for this region was in the top 56% in the North American aquaculture group. In Western Norway, the Tajima's D value for this region was at the 43rd percentile in the aquaculture group and at the 10th percentile in the wild group (Fig. 3c). These results suggest that the wild groups show signs of long-term balancing selection in the MHC region on chr14, while the aquaculture groups appear to have undergone selective sweeps since domestication began. Similar trends were observed in the 2 continents; however, likely due to demographic influences, the distribution of Tajima's D values in the control regions is generally higher in Western Norway compared to those in North America (Fig. 3c).

### Parallel Signatures of Selection in Alternate Ohnolog Copies Across Lineages

To further investigate if and how the salmonid-specific WGD contributes to the domestication of Atlantic salmon genetically, we examined whether one of the gene pairs from salmonid-specific WGD has adaptive signatures in the Western Norway aquaculture group while the other gene pair has adaptive signatures in the North American aquaculture group. By investigating XP-EHH outlier SNPs in genic regions, we detected 4 loci with ohnologous genes (i.e. genes duplicated from the WGD) under aquaculture-specific selective sweeps, 1 containing potentially multiple sweep signals. In addition to the *daxx* gene on chromosome 14, which was identified as a gene under selection in both lineages, its ohnolog on chromosome 27 (ENSSSAG00000077402) was identified as under selection in the Western Norwegian aquaculture group. In addition, we identified 3 ohnolog gene pairs under parallel domestication-related sweeps across the 2 distinct geographical regions: the bromodomain-containing 2a (*brd2a*) and proteasome 20S subunit beta 13a (*psmb13*) genes within 100 kb of *daxx* on chromosome 14 in the North American aquaculture group and chromosome 27 in the Western Norwegian aquaculture group, and ABR activator of RhoGEF and GTPase (*abr*) on chromosome 4 in the North American aquaculture group and chromosome 13 in the Western Norwegian aquaculture group (Figs. 2 and 3a and Table 2). The *brd2* gene has previously been reported as under positive selection in Atlantic salmon in aquaculture in Canada compared to their wild counterparts (López et al. 2019), which aligns with its identification as part of the selective sweep in this study.

**Table 2:** Genes under parallel selection based on the XP-EHH test

Gene ensembl ID	Gene name	XP-EHH signatures	Region	Chr	Start	End
ENSSSAG00000040701	daxx_chr14	Both_Populations	MHC	14	64,304,883	64,330,023
ENSSSAG00000040723	Tapbp	Both_Populations	MHC	14	64,331,687	64,338,464
ENSSSAG00000093386	tap2a	NAmerica	MHC	14	64,476,753	64,493,938
ENSSSAG00000085704	psmb13a_chr14	Parallel_NAmerica	MHC	14	64,463,937	64,467,854
ENSSSAG00000041842	psmb8b_chr14	Parallel_NAmerica	MHC	14	64,459,783	64,462,906
ENSSSAG00000042614	brd2a_chr14	Parallel_WNorway	MHC	14	64,524,519	64,536,467
ENSSSAG00000077402	daxx_chr27	Parallel_WNorway	MHC	27	10,402,918	10,411,454
ENSSSAG00000077187	tcf19	WNorway	MHC	27	10,323,665	10,328,897
ENSSSAG00000077419	Uba	WNorway	MHC	27	10,426,981	10,465,906
ENSSSAG00000078726	Vhsv	WNorway	MHC	27	10,801,247	10,804,941
ENSSSAG00000078638	vps52	WNorway	MHC	27	10,789,984	10,840,875
ENSSSAG00000077397	zbtb22b	WNorway	MHC	27	10,394,411	10,398,690
ENSSSAG00000077772	col11a2	WNorway	MHC	27	10,645,732	10,694,689
ENSSSAG00000077561	psmb13a_chr27	Parallel_WNorway	MHC	27	10,521,614	10,527,869
ENSSSAG00000085574	psmb8a_chr27	Parallel_WNorway	MHC	27	10,518,241	10,521,084
ENSSSAG00000077713	brd2a_chr27	Parallel_NAmerica	MHC	27	10,621,388	10,634,539
ENSSSAG00000048882	abr_chr13	Parallel_WNorway	…	13	77,743,389	77,966,280
ENSSSAG00000001855	abr_chr4	Parallel_NAmerica	…	4	56,687,253	56,919,875

We described the gene name in this table if the gene or duplicated gene pair is overlapped with 1 or more outlier SNP(s) in XP-EHH test in the Western Norwegian aquaculture group and North American aquaculture group. Start and End refer to the starting and ending locations of genes on the chromosome, respectively, based on the Ssa v.3.1 annotation on Ensembl. Duplicated genes, gray shade (see also Figs. 2 and 3) are defined according to Gillard et al. 2021.

In addition to the shared genes above, the MHC class I region (Lukacs et al. 2007, 2010) also contains salmonid-lineage-specific gene copies with overlapping XP-EHH outliers. In the Western Norwegian aquaculture group, these include *uba*, *vhsv*, *col11A2*, *tcf19*, *zbtb22b*, *psmb8a*, and *vsp52* on chromosome 27. In contrast, *the psmb8b* and *tap2a* genes on the homeolog chromosome 14 show selection signatures in the North American aquaculture group (Figs. 2 and 3a and Table 2). These lineage-specific selection patterns on homeolog regions suggest parallel selection targeting the MHC class I region.

To estimate the degree of selection signals in ohnolog genes expected by random chance, we performed a simulation analysis by randomly placing putative genes with outlier XP-EHH values across the genome 10,000 times. For cases where “one gene exhibited selection in one population while its duplicate showed selection in the other population,” we observed 4 such pairs in empirical data, with a simulated probability of 0.03 of observing 4 or more by chance. Similarly, for cases where “the same gene exhibited selection in both populations,” 2 such genes were observed, with a simulated probability of 0.027. These results indicate that while the observed selection patterns in duplicated genes are not extremely rare, they are also not entirely expected by chance.

### Expression Pattern of the Genes Under Putative Selection

We leveraged publicly available RNA-seq datasets spanning various developmental stages and tissues. This allowed us to infer the potential functions of these putatively adaptive genes and regulatory difference of homeolog gene copies.

**Fig. 4.**
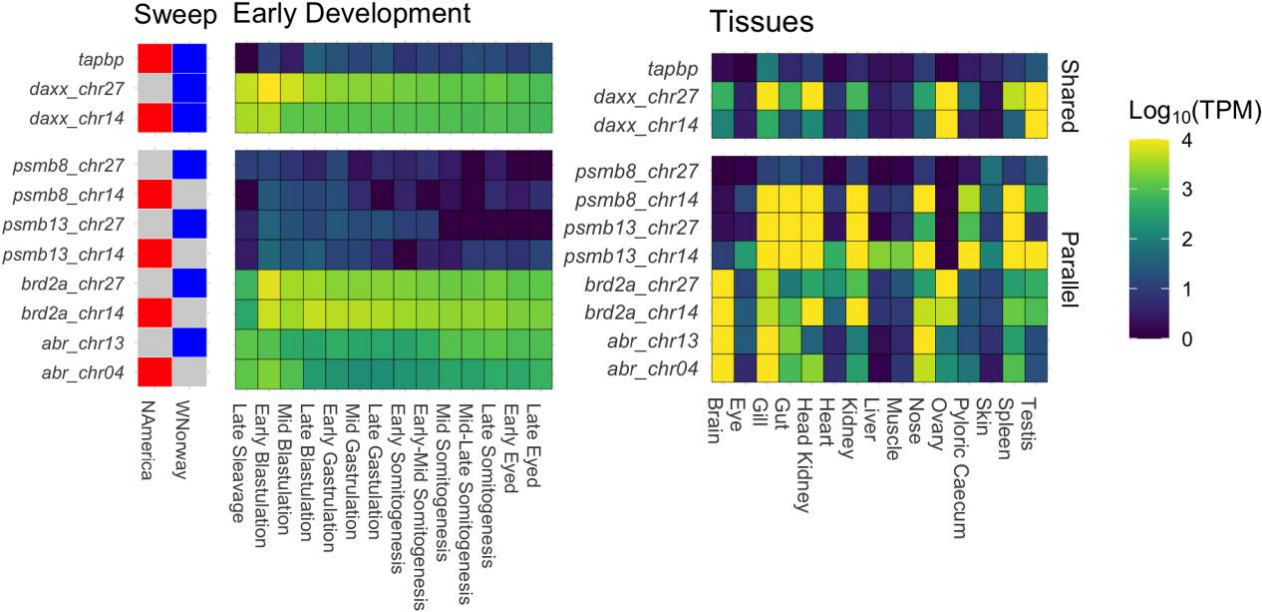
Expression of genes under parallel selection or parallel selection on the homeologous regions in early development stages and mature tissues. Gene expression is represented as log-transformed TPM. The sweep panel indicates the gene that has undergone selective sweeps in North America or Western Norway. The left heatmap shows the gene expression levels during early development, from late gastrulation to the eyed egg stage. The right heatmap shows the gene expression levels across different tissues in the mature Atlantic salmon. We used publicly available RNA sequencing data from early development stages (AQUA-FAANG project No. PRJEB51855) and from various tissues of healthy fish (NCBI Project No. SRP011583) including brain, eye, gill, gut, head kidney, heart, kidney, liver, muscle, nose, ovary, caecum, skin, spleen, and testis of Atlantic salmon in Europe.

Both *daxx* homeolog gene copies, showed high expression in the ovary, testis, and during early blastulation stages (Fig. 4), suggesting a role in both reproduction and early development. Additionally, *daxx_chr27* is also highly expressed in the gill, spleen, and head kidney, indicating its role in immunity. This pattern suggests that both copies may contribute to multiple physiological processes.

The *tapbp* gene, showed low expression in most tissues in the healthy fish. This gene was, though, moderately expressed in the gill (Fig. 4), which serves multiple important functions, including gas exchange, osmoregulation, and acting as a gateway for potential pathogens and its expression pattern suggests a potential role in the immune response (Amill et al. 2024). It is reported that *tapbp* expression is upregulated in a rainbow trout monocyte/macrophage cell line in response to viral infection (Sever et al. 2014).

Both homeologous copies of *brd2* show high expression in the brain and gill, while *brd2_chr27* is also expressed in ovary and *brd2_chr14* is expressed in head kidney, kidney, and nose (Fig. 4). As with *daxx*, this pattern indicates that each copy may have common and divergent roles. Both copies also display high expression levels during the early stages of development, particularly in early blastulation (Fig. 4). This is consistent with the established roles of *brd2* in reproductive and developmental processes, including spermatogenesis and folliculogenesis (Rhee et al. 1998), and egg polarity in zebrafish (*Danio rerio*) (DiBenedetto et al. 2008). *Brd2* is also responsible for nervous system development, morphogenesis and differentiation of the digestive tract in zebrafish (Murphy et al. 2017). In the GO analysis, both *daxx* and *brd2* appear in the organelle organization category that is enriched in genes under selection in Norwegian aquaculture (Table 1).

Another duplicate pair *Psmb8*, codes for a beta subunit of the type 8 proteasome, which facilitates endopeptidase activity and proteasomal protein catabolism (Arima et al. 2011). *Psmb8a_chr14* is highly expressed in many tissues, including immune-related tissues such as the gill, head kidney, and spleen, while *psmb8a_chr27* did not show such high expression, suggesting possible regulatory difference (Fig. 4). Both copies of *abr*, which activate cerebellar development in multiple vertebrates (Chuang et al. 1995; Mulherkar et al. 2014; Guo et al. 2020), were highly expressed in the brain (Fig. 4), also suggesting a neurological function in Atlantic salmon.

## Discussion

Domestication often involves unintentional selection for traits such as tameness, reduced stress, and changes in neurological and behavioral responses, enabling species to better coexist with humans (Fleming et al. 1996; Islam et al. 2020). In contrast, artificial selection deliberately targets desirable traits linked to dietary, economic, or social factors. Over the past 50 years (∼15 generations), independent Atlantic salmon populations have undergone both domestication and artificial selection for key production traits, resulting in specialized phenotypes significantly distinct from their wild counterparts (Milla et al. 2021). Parallel evolution across different populations is expected due to similar captive environments and selection pressures (Bourret et al. 2013; Elmer et al. 2014). Moreover, aquaculture species that experienced WGD, exemplified with salmonids, may have benefited from this genomic event, potentially hosting the genetic resources to adapt to novel, artificial environments, and intensive selection of aquaculture (Lien et al. 2016; Chen et al. 2019; Xu et al. 2019; Du et al. 2020; Redmond et al. 2023; Rondeau et al. 2023).

In this study, we investigated how domestication and artificial selection have shaped the genomic landscapes of farmed populations compared to the wild. Genome-wide scans using whole-genome sequencing data from Atlantic salmon populations in North America and Western Norway revealed evidence of genetic divergence between farmed and wild populations. We also identified parallel selection sweeps driven by positive selection in the 2 farmed strains, addressing a knowledge gap in previous research. Among these, our findings highlight parallel ohnologs—a rare case where WGD-derived homeologous regions show shared selection signals during independent domestication events.

### Parallel Selective Sweeps in MHC Genes in Aquaculture Across Continents: Likely Retention of Variation Under Long-term Balancing Selection

Our comparative study identified 82 SNPs within the *daxx* and adjacent *tapbp* genes, including 5 nonsynonymous SNPs, shared selective sweeps in aquaculture population pairs in Western Norway and North America. Both genes are members of the MHC class IA region in Atlantic salmon (Grimholt 2018), suggesting crucial roles in the immune system. The *tapbp* gene encodes a transmembrane glycoprotein, which stabilizes the peptide loading complex of MHC class I molecules, aiding in T cell immune recognition (Ortmann et al. 1997; Lehner et al. 1998; Garbi et al. 2003). The *daxx* gene has a broad range of functions in vertebrates such as regulation of telomere length and stability (Heaphy et al. 2011) and chromatin remodeling (Drané et al. 2010; Goldberg et al. 2010; Rapkin et al. 2013; Lovejoy et al. 2020). Subsequent research has shown that daxx is also involved in apoptosis (Khelifi et al. 2005; Yao et al. 2014; Détrée and Gonçalves 2019) and as a tumor suppressor (Mahmud and Liao 2019). However, its interaction with the MHC class I in fish, especially in salmon remain elusive.

Our findings suggest that long-term balancing selection has maintained high allelic diversity in the MHC regions across populations in North America and Western Norway that diverged ∼1 million years ago (Rougemont and Bernatchez 2018), as observed in other vertebrates (McConnell et al. 2016; Veríssimo et al. 2023). This diversity allows the shared, ancient SNPs in the MHC region to undergo parallel selection in aquaculture conditions. In contrast, non-MHC regions likely lack shared SNPs already due to genetic drift, which has led to fixation in each lineage and reduced the potential for parallel selection. In addition, genomic structural differences between the 2 lineages may have reduced the number of shared variants outside the MHC regions.

In natural environments, we can assume that the allelic diversity at the MHC locus of Atlantic salmon has been maintained through balancing selection. However, in aquaculture settings, specific alleles have been selected due to common selective pressures for increased immune response to infectious diseases in high-density net pen populations (Krkosek 2010). Alternatively, this selection may target other phenotypic effects of these genes exemplified by the fact that *daxx* also plays a key role in apoptosis (Michaelson et al. 1999; Khelifi et al. 2005). While nonsynonymous SNPs have been identified, pinpointing the causal SNPs needs more targeted approaches, such as MHC-specific sequencing, to fully resolve this rapidly evolving region (Sundaram et al. 2020) and functional analysis, such as gene editing. In the long term, coevolution between hosts and pathogens could lead to currently advantageous alleles becoming disadvantageous (Spurgin and Richardson 2010).

In addition, lineage-specific selective sweeps in other MHC genes further support the role of artificial selection in shaping immunity, even when the specific SNPs and genes with adaptive signatures differ between the 2 domesticated lines.

### Parallel Selection on Homeologous Regions

We observed parallel selection on 2 paired regions of salmon-specific WGD-derived ohnologs (chr14-27 and chr4-13), and one major region is the MHC region with high Tajima's D value in the aquaculture populations. This suggests balancing selection has been contributed in maintaining adaptive immune variation across duplicates in addition to prolonged tetraploid recombination, as observed in the late rediploidization LORe regions (Robertson et al. 2017). However, further in-depth research is needed to fully resolve how WGD-derived variation has contributed to adaptation, particularly in the context of domestication.

In addition, we observed 4 WGD-derived gene pairs under parallel domestication-related selection across 2 distinct geographical lineages: *psmb8*, *psmb13a*, *brd2a* on chr14 and chr27 (genes on MHC regions), and *abr* on chr4 and chr13 (gene involved in neurological and brain functions; Chuang et al. 1995; Mulherkar et al. 2014; Guo et al. 2020). Such selection signatures may be associated with ongoing adaptation to aquaculture conditions. In aquaculture environments, fish are raised under higher density conditions than in the wild, with controlled lighting, temperature, and salinity levels. In such artificial environments, advantageous alleles in captivity may differ from beneficial alleles in nature. Disease outbreaks are a major challenge in aquaculture, regardless of species (Cain 2022). Disease resistance, a key trait in aquaculture, may be enhanced by such immune genes on homeologous regions under selection. While this suggests that WGD-derived variation may contribute to specific adaptations, such occurrences are rare and its impact appears to be limited, likely due to the ancient nature of the WGD event and subsequent gene diversification or pseudogenization. Nonetheless, these genes may play a supporting role in the adaptation of Atlantic salmon to aquaculture environments. In this context, the contribution of WGD primary contributions seem to be (i) allowing for increased immune variation maintained among duplicated immune gene complexes and (ii) reducing pleiotropy through subfunctionalization (Marchant et al. 2019). While genetic changes resulting from WGD may contribute to specific adaptations, domestication has occurred across diverse phylogenetic lineages with varying genetic backgrounds, indicating that multiple genomic pathways can facilitate adaptation to human-mediated environments.

The role of WGD-derived variation in adaptation is not unique to Atlantic salmon. Parallel selection on WGD-originated gene pairs has been reported in other taxa, such as vibrant skin color in common carp and leaf-heading traits in *Brassica* species (Cheng et al. 2016; Wang et al. 2024), demonstrating that duplicated genes can contribute to adaptive evolution although the underlying genetic mechanisms may differ across taxa. Our findings provide limited direct evidence that WGD derived variation facilitated adaptation to aquaculture conditions, indicating that many other factors, including additional facets of species biology and genetics, selective breeding and environmental pressures, are also crucial in shaping domestication outcomes. Further studies integrating functional validation and broader taxonomic comparisons will be necessary to disentangle these contributions.

### Sema Genes Under Selection in the Western Norwegian Aquaculture Group

Building on understanding neural-related genetic selection in aquaculture, we underscore the sema genes (Alto and Terman 2017). GO enrichment analysis revealed that genetic SNPs under selection related to semaphorins were over-represented in the Western Norwegian aquaculture group (*sema 4ab*, *sema5ba*, *sema6d*, and *sema6e*). Semaphorin is involved in axon guidance and synapse formation (Luo et al. 1993; Jongbloets and Pasterkamp 2014). Semaphorins have also been detected under selection in previous studies of aquaculture Atlantic salmon populations (Gutierrez et al. 2016; Naval-Sanchez et al. 2020), supporting our findings. Such genes under lineage-specific selection should also contribute to the domestication of Atlantic salmon. It has been reported that structural variation showing genetic divergence between farmed and wild Atlantic salmon is linked to synaptic genes responsible for behavioral variation in Atlantic salmon (Bertolotti et al. 2020). Such work suggests that individuals with greater stress tolerance have been selectively favored in aquaculture environments. Meanwhile, it is reported that fast-growing fish tend to be more aggressive (Nicieza and Metcalfe 1999). So, it is also possible that the fast-growing fish have been selected, potentially leading to the inadvertent selection of genes associated with aggressive behaviors as a byproduct.

## Conclusion

Our findings contribute to a better understanding of the genomic mechanisms underlying aquaculture adaptation, across continents. By identifying immune and developmental genes under artificial selection, this study highlights key processes involved in the domestication of Atlantic salmon. These results also emphasize the role of genetic diversity and redundancy in shaping evolutionary trajectories and resilience to challenges in aquaculture-specific environments.

## Materials and Methods

### Studied Populations

The present study involved 370 Atlantic salmon individuals from 2 wild and aquaculture populations across distinct phylogeographical lineages, aiming to compare genomic landscapes of domestic strains with local wild counterparts. Whole-genome sequencing data was retrieved from publicly available datasets.

We used 80 North American aquaculture samples derived from the Gaspe, St. John, and Penobscot Rivers (NCBI PRJEB34225) and 112 Norwegian MOWI strain samples from the River Bolstad, Årøy, and marine captures near Osterfjord and Sotra (NCBI PRJEB47441; Glover et al. 2009). Wild counterparts included 80 North American individuals from 8 Quebec rivers and 98 Norwegian individuals from 17 rivers in Western Norway (NCBI PRJEB38061; Bertolotti et al. 2020), see Fig. 1; supplementary tables S1 and S2, Supplementary Material online.

In North America, aquaculture samples were taken from Gaspe (*n* = 16), St. John (*n* = 53), and Penobscot Rivers (*n* = 11). Wild samples were from 8 rivers: Malbaie Charlevoix, Bonaventure, Petite rivière Cascapedia, Laval, De la Chaloupe, Jupiter, Du Vieux Fort, and Saint-Paul (*n* = 10 each). In Western Norway, aquaculture samples were from the MOWI Norwegian breeding line (*n* = 112). Wild counterparts were from 17 rivers: Vorma, Suldalslågen, Vikedalselva, Etneelva, Eidfjordvassdraget, Oselva i Os, Loneelva i Osterøy, Flåmselva, Lærdalselva, Årøy, Daleelva, Flekkeelva, Nausta, Jølstra, Oselvvassdraget, Ryggelva, and Gloppenelva (ranging from *n* = 2 to *n* = 10, supplementary tables S1 and S2, Supplementary Material online).

The North American lineages originate ostensively from single rivers and so should not have an admixed founding population; however, there is documented supplementation from Canadian rivers early in the domestication history into Maine. These lineages also show evidence of European admixture despite use of European fish in Canadian aquaculture never having been approved (Bradbury et al. 2022). The origins of domesticated North American salmon strains are the Penobscot River strain (Gao et al. 2023), the St. John's River strain (Liu et al. 2017) and the Grand Cascapedia River (Nash and Waknitz 2003). These strains originated from the Gulf of Maine, the Bay of Fundy, and the Gaspé Bay, Quebec, respectively. Sampled populations were primarily from Quebec including the Petite Cascapedia River, which neighbors the source population of the Gaspé strain (Fig. 1; supplementary tables S1 and S2, Supplementary Material online). Historically, the Gaspé and Miramichi Rivers were used for population supplementation in Maine during the 1920s–1960s. This provides a basis for comparing Canadian wild populations with domesticated strains, as the Penobscot strain originated after Canadian stock introductions (FWS Training Center: https://trainingcenter.fws.gov/resources/knowledge-resources/salmon/asalmon4.html). The admixture history of North American domesticated lineages is evident in the PCA plot (Fig. 1).

The European lineage we analyzed, MOWI, is derived from a mixture of west coast lineages, primarily from the Vosso drainage basin and the River Årøy, in the 1960s. The wild sampled rivers include the source population River Årøy in Western Norway. The Vosso population has collapsed since salmon were taken from it for aquaculture; it is highly disturbed by introgression from farmed escapees, which is high in part because of population collapse from multiple other anthropogenic disturbances. The loss of the original Vosso source population due to farm-wild introgression is well-documented. Sægrov et al. (1997) reported that escaped farmed Atlantic salmon replaced the original stock in the River Vosso (Sægrov et al. 1997). Similarly, Glover et al. (2012) provided a spatio-temporal analysis of Atlantic salmon population genetic structure throughout Norway (Glover et al. 2012). Sampling efforts include Årøy, which was used as part of the wild dataset alongside other west coast populations. Vosso, however, was excluded due to its near-total replacement by aquaculture escapees (Våge 1995). As a result, contemporary samples from Vosso cannot, unfortunately, be used as representative of the original source population. A sample of 16 other west coast populations in the surrounding region was used in place of this source population.

The founding population size of aquaculture groups is unknown, though it is assumed that there will have been a population bottleneck at the founding of the domesticated lineages. However, this will have been countered by the admixture during the founding of the European lineage and the supplementation of domesticated lineages in Maine with Canadian and likely also European salmon. Resulting in domesticated lineages that have a history of population bottlenecks, admixture, and strong artificial selection. Disentangling these processes from the data will be complex without historical samples. The influence of admixture can be seen in the wider spread of domesticated lineages in PCA space (European on PC2 and North American across PC1 and PC2, see Fig. 1) compared to the wild lineages used. The Domesticated lineages cover greater PCA space than the combined spread of the wild populations in both continents, suggesting admixture is more important than population bottlenecks. Despite the Gaspé lineage have the closest source population sampled; this is actually the furthest in PCA space from the cluster of wild populations. The North American wild populations cluster tightly and overlap with the Maine and Bay of Fundy domesticated lineages, probably reflecting the use of Canadian salmon to supplement early aquaculture further south.

### Data Preparation

Mapping, SNP calling and quality control were performed independently for each of the 4 datasets (Aquaculture Norwegian, Wild Norwegian, Aquaculture North American, and Wild North American) using the same pipeline and parameters. Illumina reads were mapped to the Atlantic salmon genome (Ssal_v3.1; GCA_905237065.2; Stenløkk 2023) using bwa-mem2 (Li and Durbin 2009). Duplicate reads were marked with gatk4-spark:4.3.0.0 MarkDuplicates. Genetic variation was identified using gatk4-spark:4.3.0.0 HaplotypeCaller with default parameters and the individual genotypes were merged with gatk4-spark:4.3.0.0 GenotypeGVCFs. To avoid the detection of false positives, SNPs were filtered by the following parameters with gatk4-spark:4.3.0.0 (McKenna et al. 2010) : « QD < 2.0 » which filters out SNPs with a low-quality score by depth of coverage; « QUAL < 50.0 » to sort SNPs with a low-quality score; « SOR > 4.0 » that removes SNPs where the number of reads supporting the reference allele is significantly skewed toward 1 strand (forward or reverse) compared to the other; « FS > 60.0 » to filter out SNPs whose reads supporting the reference allele versus the alternative allele are significantly different between the 2 strands; and finally « ReadPosRankSum < -8.0 » to remove SNPs with a low score for the difference between the mean position of the reference allele and the alternative allele among the reads supporting the SNP (https://gatk.broadinstitute.org/hc/en-us). From this large pruning, we kept only biallelic markers (SNPs) with depth >4 and <30, confident call rate >90% (GQ) and missingness lower than 10% using VCFtools (Danecek et al. 2011). The whole analytical pipeline is summarized in supplementary fig. S1, Supplementary Material online.

### Population Structure Analysis

We used 2 clustering methods to investigate genetic structure among populations and have a broad representation of the genetic differentiation between North American and West Norwegian lineages. Additional filtering steps specific to these analyses were applied to remove loci that deviated from Hardy–Weinberg equilibrium, loci with a minor allele frequency (MAF) below 5%, and linked SNPs using PLINK v1.9 (Purcell et al. 2007). Linkage disequilibrium pruning was performed using a sliding window approach (i.e. 50 SNP windows, 10 SNP step size, and an R² threshold of 0.5). We ran ADMIXTURE (Alexander et al. 2009; Alexander and Lange 2011) with a number of ancestral populations set from 1 to 20 (K). The optimal K was selected based on the lowest cross-validation error. We then conducted a PCA using PLINK v1.9 (Purcell et al. 2007) on the same filtered dataset. The results of both analyses (i.e. eigenvectors and genetic variation from PCA; genetic components from ADMIXTURE) were visualized in R 4.2.2 using the *ggplot2* (Wickham 2011) and *pophelper* (Francis 2017) R packages.

### Detection of Selection Sweeps

For the detection of putative genomic regions under artificial selection in domestic strains, we applied a genome scan using 3 statistical methods based on (i) population differentiation (F_ST_; Weir and Cockerham 1984); (ii) site frequency (Tajima's D; Tajima 1989), and (iii) cross-population test of extended haplotype homozygosity (XP-EHH; Sabeti et al. 2002). These tests were first carried out to compare aquaculture and wild populations from the same geographical region independently in order to detect lineage-specific selection signatures. Then, we investigated whether some genomic regions putatively under selection are shared by populations of different origins, indicative of parallel evolution. In the context of domestication, we expect 2 main scenarios in terms of selective sweeps. A hard selective sweep occurs when a beneficial allele arises and rapidly reaches fixation within a population. There is little genetic variation surrounding the selected allele because the advantageous SNP quickly “sweeps” through the population, leaving little time for new mutations or recombination to occur. As a result, the genetic diversity around the selected site is reduced, and the favored allele is present on a single haplotype in the population. A soft selective sweep occurs when multiple beneficial alleles, either derived from different mutations or standing genetic variation, rise in frequency or are favored within a population. During a soft sweep, there can be multiple haplotypes carrying different advantageous alleles and the genetic diversity around the selected site is not as reduced as in a hard sweep, and multiple haplotypes may show evidence of positive selection.

First, F_ST_ identifies selection using differences in allele frequencies between populations, here between domesticated and wild populations from each geographic area independently. These statistics were calculated according to the pairwise Weir and Cockerham's (1984) estimator using VCFtools (Danecek et al. 2011) in 20,000 bp nonoverlapping sliding windows using wild individuals as the reference population. We extracted the top 1% values as outliers.

Second, Tajima's D assumes that neutral genetic variation is maintained by a balance between mutation, genetic drift, and gene flow. The test is computed as the ratio between the number of segregating sites and the average pairwise nucleotide differences. Deviations from this neutral evolution can indicate putative selection or demographic events. Markers under selection can be expressed as positive and negative values, while zero values indicate a putative absence of selection. A significant positive Tajima's D value may indicate an excess of intermediate-frequency alleles, which could be caused by balancing selection, population subdivision, or recent population expansion. A significant negative Tajima's D value may indicate an excess of low-frequency alleles, which could be caused by positive selection, population bottlenecks, or purifying selection. In our study, Tajima's D was conducted on the 2 aquaculture populations independently by nonoverlapping sliding windows of 20,000 bp. As the domestication involved bottlenecks and focus on directional selection, we are more interested in negative values. The lowest values at the 1% level were taken as the significance level. F_ST_ and Tajima's D were calculated from vcf files resulting from the soft filtering (i.e depth, low quality SNPs, and missingness; see *Data preparation* section).

Finally, we investigated patterns of extended haplotypes between pairs of populations using the *rehh* R package (Gautier et al. 2017). Haplotype analyses are based on extended haplotype homozygosity (EHH) that represents the model of a selective sweep in which an adaptive de novo mutation appears on a haplotype and rapidly evolves toward fixation, thus reducing diversity around the locus (Sabeti et al. 2002). A hard sweep induces a signal of high haplotype homozygosity to be observed extending from an adaptive locus as strong rapid selection causes strong linkage that is not broken by recurrent mutation or recombination. In other words, under conditions of positive selection, a beneficial new allele can rapidly increase in frequency and create long stretches of homozygosity, or identical copies of the haplotype, around the selected site. This process results in longer and linked haplotypes detectable by EHH methods. In our case, we applied a cross-population (XP-EHH) test that compares the distribution of haplotype homozygosity between aquaculture and wild populations. When there is divergent selection between the 2 environments (wild and aquaculture), the allele selected in the focal population (aquaculture) will be on a longer haplotype. This signal will not be present when selection is concordant between the 2 environments. Negative scores of XP-EHH suggest that selection occurred in the reference population, whereas positive scores show selection in the domestic population. Unlike Tajima and F_ST_, these tests cannot directly handle SNP genotypes because they require haplotypes. Added to the soft filtering, SNPs with MAF below 5% were removed. The remaining SNPs were phased to infer the haplotypes of each population using BEAGLE v4.1 (Browning and Browning 2007) with a window size of 10,000 and 600 bp overlap. One-sided *P*-values were obtained as −log10(1-Φ[XP-EHH]), where Φ(XP-EHH) represents the Gaussian distribution function for the statistic. FDR test was performed to estimate the number of false positives and determine the significant threshold. We used −log10(*P*-value) = 4 (alpha = 0,0001) as a threshold to define significant values of XP-EHH. As XP-EHH searches for the extended haplotype, which is typical of a hard sweep, this method will be more sensitive in detecting hard sweeps than soft selective sweeps.

### SNP Annotation

Significant windows or SNPs from the 3 comparative analyses were intersected using BEDtools (Quinlan 2014) to examine markers detected by multiple methods and thus increase the repeatability of the results. For each geographical region, 3 intersections were tested: (i) F_ST_/Tajima's D, (ii) F_ST_/XP-EHH, and (iii) Tajima's D/XP-EHH. Candidate SNPs detected by 2 or more methods were then intersected with annotated genes in the Atlantic salmon reference genome (Ssal_v3.1; GCA_905237065.2 ; Stenløkk 2023) using BEDtools window to identify SNPs under selection that are contained in the same windows as the genes or that are directly mapped to genes. The overlapping genes were input into ShinyGO 0.77 (Ge et al. 2020; http://bioinformatics.sdstate.edu/go/) to extract biological functions and enrichment. All the intersects were tested independently to observe population-specific signatures. We then tested parallel evolution using only the significant SNPs from XP-EHH, as this was the most suitable method. Categories were extracted where the enrichment FDR was <0.05, Fold Enrichment >2, and the GO category contained ≥3 genes (Table 1).

### Gene Expression Analysis

We used available RNA sequencing data online (i) from early development stages (AQUA-FAANG project No. PRJEB51855) and (ii) from tissues (NCBI Project No. SRP011583) including brain, eye, gill, gut, head kidney, heart, kidney, liver, muscle, nose, ovary, caecum, skin, spleen, and testis. Data were quantified using the latest version of the Ensembl transcriptome (Ssa v.3.1). The fastq files were first filtered using fastp v0.23.2 (Chen 2023) with default settings in order to remove potential sequencing adaptors in the reads. Then, the reads were mapped using the lightweight mapper salmon v1.5.2 (Patro et al. 2017), which tracks, by default, the position and orientation of all mapped fragments. This information is used in conjunction with the abundances from online inference to compute per-fragment conditional probabilities (Patro et al. 2017). The produced TPM (transcripts per million) was visualized using the package *ggplot2* in R.

### Test for Balancing Selection on the MHC Region on Chromosome 14

Investigation of balancing selection was carried out using Tajima's D (Tajima 1989). Tajima's D value was computed in the MHC region on chromosome 14 between 64,320,000 and 64,340,000 bp. Then, Tajima's D was computed on 500 windows of 20,000 pb with more than 15 SNPs, randomly selected across the genome. We used variant effect predictor (McLaren et al. 2016) to predict SNPs consequences on coding genes.

## Supplementary Material

evaf063_Supplementary_Data

## Data Availability

Script used for the analyses in this work are available at https://github.com/paulinebuso/ATLANTIDES_pipelines
