# Supplementary material for: Parallel Selection in Domesticated Atlantic Salmon from Divergent Founders Including on Whole-Genome Duplication-derived Homeologous Regions
Source: Genome Biol Evol. 2025 Apr 18;17(4):evaf063. doi: 10.1093/gbe/evaf063 (PMC12006720; doi:10.1093/gbe/evaf063)
Supplement: evaf063_Supplementary_Data [file evaf063_supplementary_data.zip › S_figuresR.docx]

**
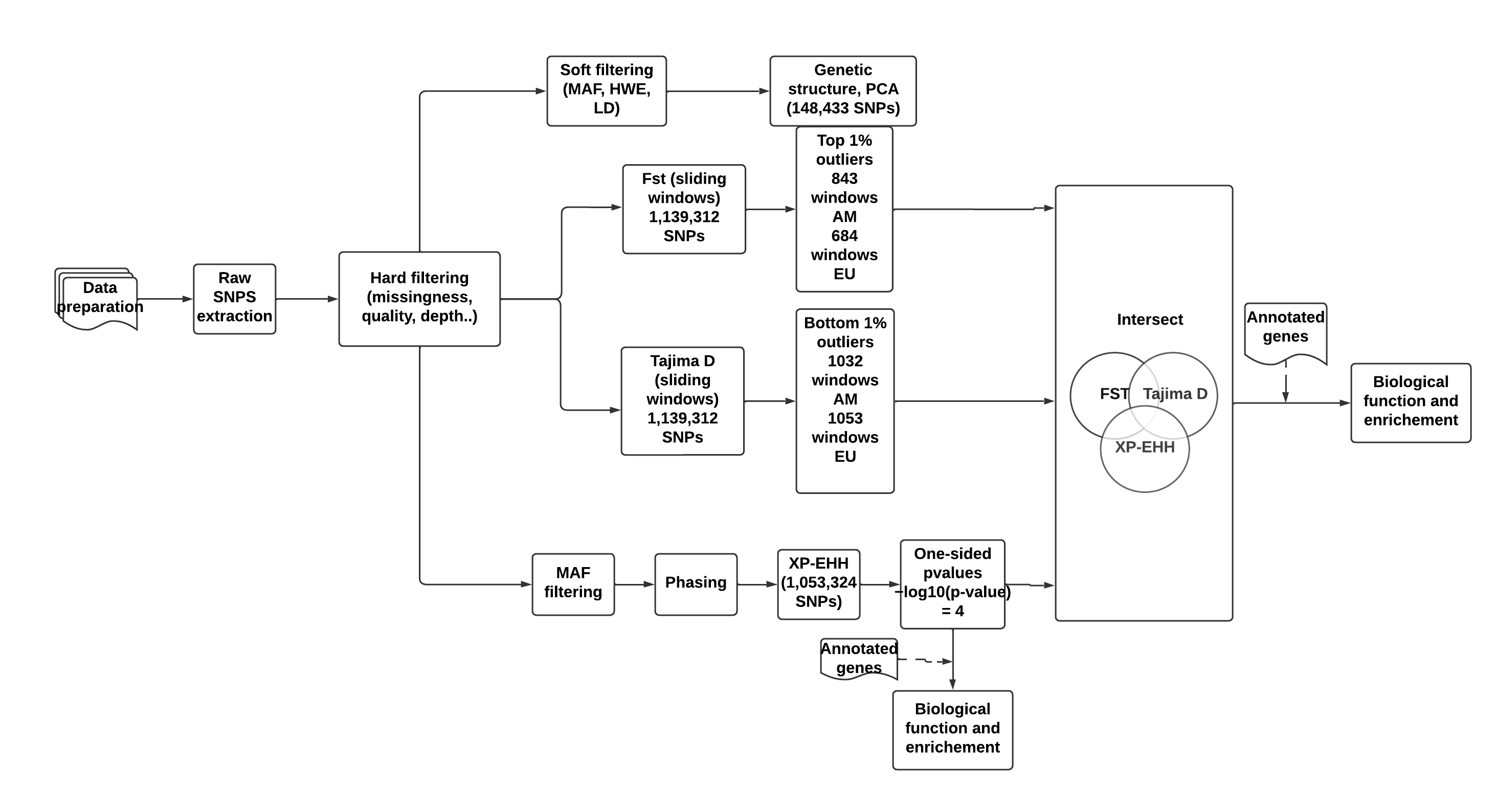
Figure S1 Methods summary**

**
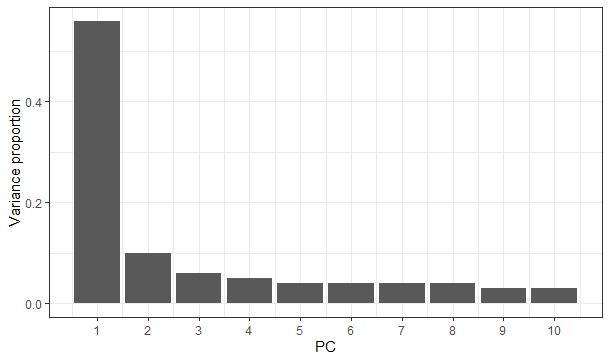
**

**Figure S2.** Genetic variance proportion distributed alongside the 10 PCs of the Principal Component Analysis (PCA).


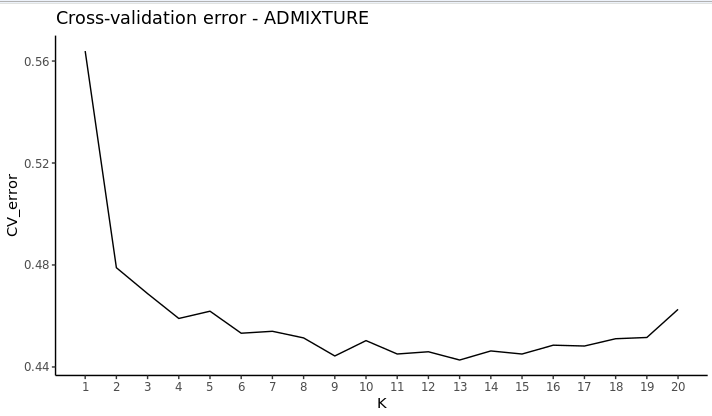


**Figure S3.** Cross-validation errors of the 20 ancestral components (K) tested with ADMIXTURE.


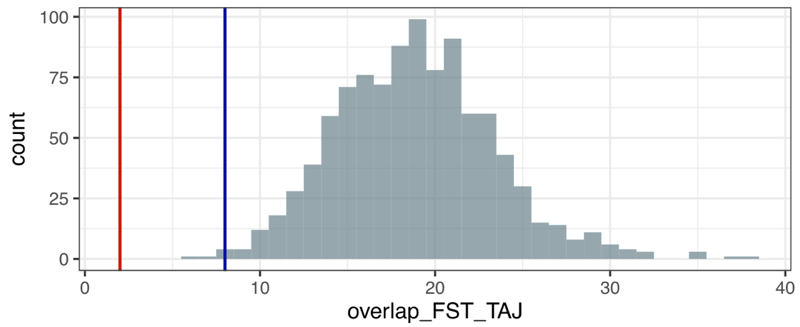

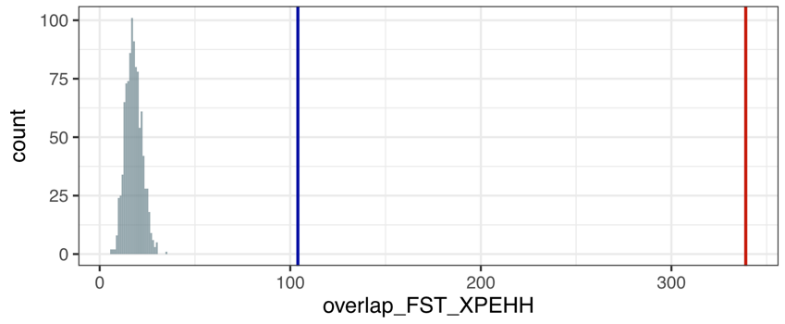


**Figure S4** Random size-matched window sampling simulations to investigate the likelihood of overlapped window discovery by multiple selection scans(n = 1000). The two are expected distributions (histogram in blue gray) after 1000 randomly selected windows in the genome vs empirically observed number of window overlapping (in red line: North America; in blue : Norway). Top plot: Overlap between Tajima’ D and FST. Bottom plot: overlap, XPEHH and FST, respectively.


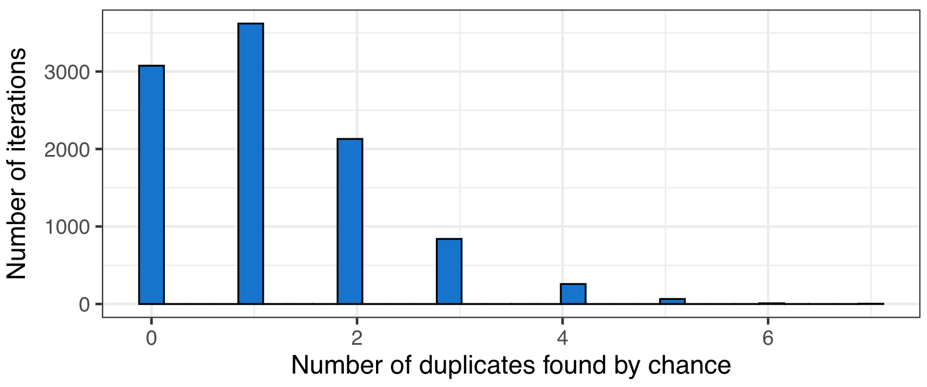


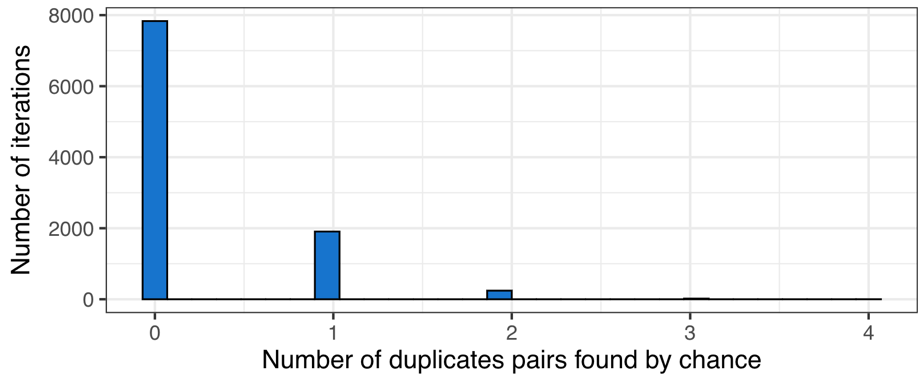


**Figure S5. Simulation results for the likelihood of observing ohnologues under selection**

We simulated hypothetical genes with outlier XP-EHH values (based on the number of empirically observed genes) for 10,000 times. For the case where "one gene showed a selection signal in one population and its duplicate showed a selection signal in the other populations, (top panel)", we observed 4 such pairs in real data, with a simulated probability of 0.03 for observing 4 or more pairs by chance.

We also simulated cases where "the same gene showed selection signals in both populations, (bottom panel)”. In this case, we observed 2 such genes, with a simulated probability of 0.027 for observing 2 or more pairs by chance.


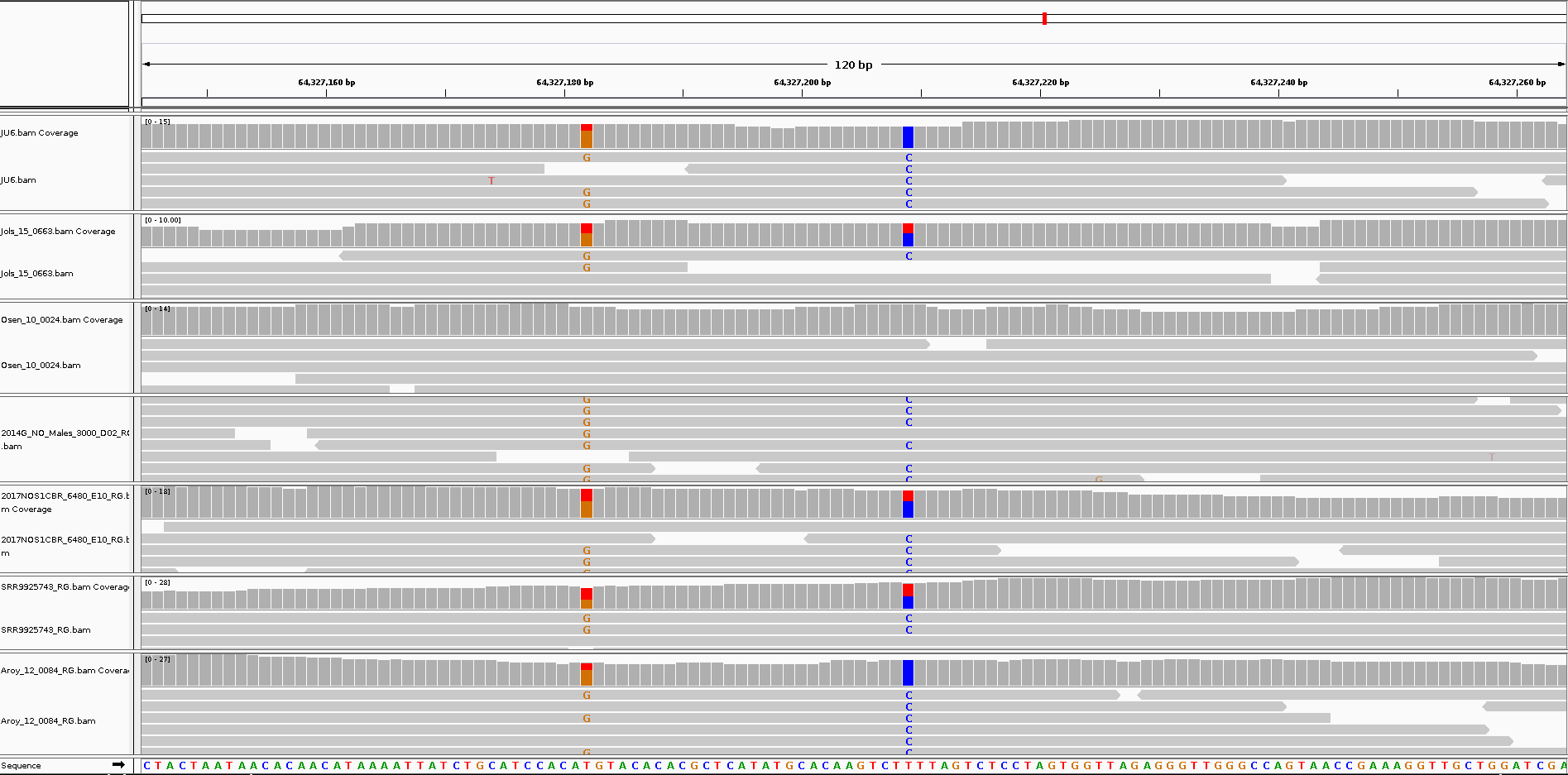


**Figure S6. IGV: Integrative Genomics Viewer screenshot of SNPs from 7 individuals from the four populations**

SNPs are genotyped at site 6 237 182 and 6 327 209 in the VCF file and are indeed observed at these positions in IGV.
